# Intelligent biology and medicine: Accelerating innovative computational approaches

**DOI:** 10.1016/j.csbj.2024.11.044

**Published:** 2024-11-28

**Authors:** Fuhai Li, Li Liu, Kai Wang, Xiaoming Liu, Zhongming Zhao

**Affiliations:** Institute for Informatics, Data Science and Biostatistics (I2DB), Washington University in St Louis, St Louis, MO 63108, USA; Department of Pediatrics, Washington University in St Louis, St Louis, MO 63108, USA; College of Health Solutions, Arizona State University, Phoenix, AZ 85004, USA; Biodesign Institute, Arizona State University, Tempe, AZ 85281, USA; Raymond G. Perelman Center for Cellular and Molecular Therapeutics, Children's Hospital of Philadelphia, PA 19104, USA; Department of Pathology and Laboratory Medicine, University of Pennsylvania, Philadelphia, PA 19104, USA; USF Genomics & College of Public Health, University of South Florida, 3720 Spectrum Boulevard, Suite 304, Tampa, FL 33612, USA; Center for Precision Health, McWilliams School of Biomedical Informatics, The University of Texas Health Science Center at Houston, Houston, TX 77030, USA

**Keywords:** Algorithm, Artificial intelligence, Cancer informatics, Computational tool, Viral infection

## Abstract

In this editorial, we summarize the 2023 International Conference on Intelligent Biology and Medicine (ICIBM 2023) conference which was held on July 16–19, 2023 in Tampa, Florida, USA. We then briefly describe the nine research articles included in this special issue. ICIBM 2023 scientific program included four tutorials and workshops, four keynote lectures, four eminent scholars’ presentations, 11 concurrent scientific sessions, and a poster session. We had total of 88 scientific oral presentations, including 62 regular oral presentations and 26 flash talks, as well as 46 poster presentations. The topics of these presentations covered artificial intelligence (AI), data sciences, bioinformatics, computational biology, genomics, biomedical informatics, among others. This special issue included nine peer reviewed manuscripts selected from those submitted to our conference. These articles cover a range of topics on the methods and applications of ML and AI models in the biomedical domain.

## 1 Introduction

The 2023 International Conference on Intelligent Biology and Medicine (ICIBM 2023), the official conference of the International Association for Intelligent Biology and Medicine (IAIBM), was successfully held on July 16–19, 2023 at Tampa, Florida, USA. Since its inception in 2012, the ICIBM conference series has become a forum to promote innovative research, interdisciplinary discussion, educational opportunities, and collaborative efforts among the fields of artificial intelligence (AI), data science, bioinformatics, systems biology, biomedical informatics, among others.

Building upon the successes of the conferences in the previous years[1], [2], [3], [4], [5], [6], [7], [8], ICIBM 2023 continued to provide an enriched scientific and educational program to the intelligent biology and medicine communities. ICIBM 2023 scientific program included four tutorials and workshops, four keynote lectures, four eminent scholars’ presentations, 11 concurrent scientific sessions, and a poster session. We had total of 88 scientific oral presentations, including 62 regular oral presentations and 26 flash talks, as well as 46 poster presentations. ICIBM 2023 also included four special sessions: “Technology Session”, “Dynamics of Transcriptional Regulation Towards Single Cell, Single Molecular, and Spatial Omics”, “Artificial Intelligence on Big Data: Promise for Early-stage Trainees” (13 trainees), and “Special Topics on Genomics and Translational Bioinformatics.” The topics of these presentations included deep learning and machine learning, AI in translational research, applications of GPT and large language models, next-generation sequencing, single-cell omics and spatial omics, microbial informatics, medical informatics, public health informatics, pharmacoinformatics, cancer informatics, integrative genomics, complex genetics studies, multi-modality, statistical genomics, and other omics research, computational methods and novel applications of computational tools, and others. Realizing the prominent impact of AI and data science, ICIBM spanned current topics for machine learning and deep learning, with the intention of bridging topics together and fostering a cross-disciplinary environment. The 11 scientific presentation sessions included the speakers that were peer-review selected (typically two to three program committee members for each paper) from the top-ranked manuscripts and abstracts, spanning a wide array of expertize including data science, AI, bioinformatics, genomics, next-generation sequencing analysis, cancer informatics, medical genomics, and computational drug discovery.

In 2022, ICIBM first time added a special session “Artificial Intelligence on Big Data: Promise for Early-stage Trainees”, which promoted the college and high school students to participate and interact with senior scientists through ICIBM. We continued on this special session, with 13 early-stage trainees presented in this session. This session has been very popular, gaining the support from not only the conference attendees, but also the parents of some of these trainees. The impact expanded to the general society and community, which highlights the importance of such event to the general public. The poster session had a total of 46 poster presentations. The four tutorials and workshops were: “Tutorial on Collection and Analyses of Long-Read Transcriptome and Epitranscriptome Data”, “Workshop on Applications of AI in Translational Research”, “Workshop on Microbiome Data Analysis”, and “Workshop on Prompt Bioinformatics – Application of ChatGPT and Large Language Models”. ICIBM 2023 had an excellent line-up of four world-renowned keynote speakers: Dr. Yidong Chen from The University of Texas Health Science Center at San Antonio, Dr. Bradley Malin from Vanderbilt University, Dr. Brooke Fridley from Moffit Cancer Center, and Dr. Jeffrey Townsend from Yale University. They shared their foundational works and perspectives in (1) deep learning cellular responses towards cancer drug response, (2) building ethically viable biomedical data science environments, (3) analytical strategies for unveiling the tumor immune microenvironment using spatial transcriptomics, and (4) evolution models and applications in cancer.

Below, we briefly summarize the six research articles selected for this special issue.

## 2 Summaries of manuscripts in this issue

Below, we summarize the contribution of nine papers included in this special issue: “Intelligent Biology and Medicine”. These papers cover a wide spectrum of topics in computational analysis methods and AI models for omic and image data analysis.

In the first paper, Chandrashekar et al. developed a novel multi-view attention-based deep neural network model, DeepCORE[9], to model the relationship between genetic, epigenetic, and transcriptional patterns and identifies co-operative regulatory elements (COREs). In addition to the improved prediction accuracy, it contains an interpreter that can extract the attention values embedded in the deep neural network, and map the attended regions to putative regulatory elements, and infers COREs based on correlated attentions. The valuation results using transcriptomes of multiple tissues and cell lines showed DeepCORE outperformed the state-of-the-art methods.

Tumor evolution using single-cell sequencing technologies is important for understanding the somatic variation of cancer cells. Reliable inference of the evolutionary relationship is a critical step for evaluation analysis. To tackle the errors and missing bases in single-cell sequencing, in the first paper, Miura et al. [10] developed a computational model integrating standard phylogenetic optimality principles and patterns of co-occurrence of sequence variations to generate expansive and accurate evolution phylogenies using single-cell sequence datasets. The model can reconstruct recurrent mutations, mutational reversals and phylodynamics to infer metastatic cell migrations. In addition, the model can be used for analyzing CRISPR/Cas9 genome editing datasets.

Mutation density analyses is important in cancer research. In addition to the mutation density analysis in protein-coding regions, Zhang et al. [11], for the first time, analyzed the mutational density patterns of long non-coding RNA (lncRNA) of 57 unique cancer types using 80 cancer cohorts. The analysis results showed that the comparable patterns between protein-coding mRNA and lncRNA. Moreover, the quantified transcription strand biases with a Log Odds Ratio metric showed that some of these biases are associated with patient prognosis. The analysis results illustrate lncRNA’s potential roles in cancer research.

Cancer cell lines are essential in cancer research and drug discovery. To avoid the use of wrong cancer cell lines, many journals require cell line authentication in their articles. To facilitate the cancer cell line authentication, Bu et al. developed a novel computational model and tool, named CCLHunter[12]. Specifically, the model makes use of the single nucleotide polymorphisms, expression profiles, and kindred topology to authenticate human cancer cell lines accurately. The tool is accessible via standalone software and a web server.

Cancer is a complex disease, which is affected by a set of genetic and environmental factors. Bai et al. [13] creatively integrate the omic data from The Cancer Genome Atlas (TCGA) and geospatial environmental risks of the registry hospitals from the United States Environmental Protection Agency (USEPA), and explored mutational signatures from the aspect of racial disparity and pollutant exposures, as well as the hepatitis B and C viruses and human papillomavirus infection statuses. The results showed that mutation frequencies of key oncogenic genes varied substantially between different races; the increased pollution level is associated with worse survival. Moreover, it is found that the aflatoxin mutational signature was exacerbated by hepatitis infection for Asian patients but not for White patients. In summary, the analysis identified that the environmental pollutant exposures can increase mutational level, and cause poor cancer prognosis.

In single cell sequencing, sample multiplexing is an important strategy to reduce costs and minimize batch effects by pooling multiple samples together. Demultiplexing is critical for multiplexing data analysis by assigning cells to individual samples. In this study, Huang et al. [14] developed a novel demultiplexing model, scDemulitplex, which makes use of the beta-binomial distribution to model hashtag oligo counts and uses an iterative strategy for model refine. In the comparison results, scDemultiplex achieved better performance in both high-quality and low-quality data, compared with seven widely used demultiplexing approaches.

Using scRNA-seq data of cancer samples, differentially expressed genes in diverse cell types can be identified to understand the cell type-specific dysfunctional signaling pathways. Ruan et al. [15] conducted an integrative analysis of two scRNAseq dataset of papillary thyroid carcinoma (PTC) to identify the consistently expressed genes across 32 cell types. In the results, 31 consistently differentially expressed genes across seven cell types, i.e., B cells, endothelial cells, epithelial cells, monocytes, NK cells, smooth muscle cells, and T cells, were identified. The functional enrichment analysis using these gene biomarkers showed the dysfunctional adaptive immune response. Moreover, these genes are associated with the disease-free survival. Among these genes, KRT7 was further identified as a potential target that promotes thyroid cancer metastasis through the epithelial-mesenchymal transition and NF-κB signaling pathway.[16].

The study of functional effect of amino acid variants is a critical biological problem. However, it is expensive to experimentally study functional effects of amino acid variants for a large number of proteins. To tackle this problem, Derbel et al. [16] developed a novel deep learning model, Rep2Mut-V2, in which the learned representation from transformer models were integrated. Compared with the state-of-the-art methods, the model significantly improved the prediction accuracy for 27 types of measurements of functional effects of protein variants. The Rep2Mut-V2 can facilitate in the interpretation of variants in human disease studies.

Caenorhabditis elegans (C. elegans) have been used for studying lifespan. Automatic quantitative analysis of C. elegan images is important for the analysis. In this study, Garvi et al. [17] developed a novel AI model for automatic quantitative analysis of live C. elegan images. The model consists of two steps: the C. elegans are first detected by using a Faster R-CNN model; and then a regression neural network is used to predict the count of live nematodes on each day of the experiment. The results showed the robust lifespan analysis using petri dish images of live C. elegans.

## Author statement

All authors approved the manuscript contents and affirmed that this work is not under consideration or published elsewhere. We have no other submission that contains overlapping content with this manuscript. We claim no conflict of interest.

## Credit authorship contribution statement

F.L, L.L, K.W, X.L, Z.Z wrote and edited the manuscript. All authors read and approved the final manuscript.

## Declaration of Competing Interest

All authors claim no conflict of interest.
